# Trends of the journal: letters, reviews, abstracts, and manuscripts from abroad

**DOI:** 10.1590/S1980-57642009DN20200001

**Published:** 2008

**Authors:** Ricardo Nitrini

**Affiliations:** Editor-in-Chief

This issue of **Dementia & Neuropsychologia** inaugurates a new section: Correspondence, containing comments and criticisms from our readers on both articles and the editorial approach. This tends to be one of the most interesting sections in many periodicals, scientific or otherwise, and we hope this will also be the case here.

**Views & Reviews** is another section available to readers as a forum for comments on important themes or news that deserve dissemination to the scientific community. It is a section also devoted to reviews, which is now a key focus in that we want to give increasing weight to our reviews.

A scientific periodical has many functions and targets besides the most obvious of publishing new and important scientific advances. An objective we have already mentioned is the role of establishing contact between researchers and the scientific community. Another aim which is especially relevant is to keep readers abreast of the latest developments, particularly because it is very difficult for books to fulfill this role in this era of rapid advance in science. Reviews by eminent researchers, given freedom to express their views on subject in which they have expertise, are also valuable to both younger and more experienced readers alike. One could argue that the majority of scientific periodicals publish excellent reviews, often written by renowned researchers from different areas, which may at first suggest that to publish such reviews in **Dementia & Neuropsychologia** could be a fruitless exercise of no interest to our readers. This is not a correct assumption however, as there are many themes with special interest to developing countries or Latin America that are seldom the focus of reviews, thus remaining unknown to most researchers, even by those doing research in the specific field. The paucity of reviews on these themes contributes to ostracize many papers published in less well-known journals, causing duplication of research or reiteration of methodological problems recognized in former studies. The numerous themes of great interest to research or practical activity in developing countries identified thus far, coupled with the level of acceptance of our invited authors have increased our confidence that this strategy will prove important for **Dementia & Neuropsychologia** in its endeavor to carve out a niche among leading journals in the field in the coming years.

Once again we are publishing the abstracts of the Theses in Neurosciences entered for the best theses awards presented in Brazil last year, in a contest sponsored by the 4^th^ Congress of Brain, Behavior and Emotions, held in Bento Gonçalves, Rio Grande do Sul, in May 2008. This is one of the most interesting meetings of basic and clinical neuroscientists, involving other professionals more linked to clinical practice, which brings together a range of professionals from psychoanalysts to bench researchers to the same round table (a large couch, as a matter of fact). The evident difficulty in maintaining a high level of interaction among such diverse points of view has been overcome by the extreme creativity of the organizers, who always surprise the participants with the unusual characteristics of the themes or debates. The opportunity to again publish these theses is both welcome and valuable.

The first papers from abroad published in our journal were submitted by Professor Helmut Heinsen from Wuerzburg, Germany, who was working with Lea Grinberg, a Brazilian neuropathologist,^1^ and by the group lead by Professor Kenichi Meguro, from Tohoku University, Sendai, Japan.^2^ In this issue we publish an article by Professor John Hodges’ group,^3^ formerly at Cambridge but now in Sydney, Australia. In his letter, Prof. Hodges, who is one of the most renowned researchers in the field of frontotemporal lobar degenerations, stated that the main reason for submitting the manuscript to **Dementia & Neuropsychologia** was to better disseminate the Cambridge Behavioural Inventory Revised to the Latin-American community. However, we read between the lines that another reason, which also influenced Heinsen and Meguros’ decision, was to give support to our journal. Indeed, we feel that providing wider access to the Latin-American community is one of the differential characteristics offered by our journal, a special feature that we hope will be recognized by our future contributors from the international community.
